# Biallelic variants in *RINT1* present as early-onset pure hereditary spastic paraplegia

**DOI:** 10.1172/JCI178919

**Published:** 2024-07-11

**Authors:** Vicente Quiroz, Laura Planas-Serra, Abigail Sveden, Amy Tam, Hyo-Min Kim, Umar Zubair, Dario Resch, Afshin Saffari, Matt C. Danzi, Stephan Züchner, Maya Chopra, Luca Schierbaum, Aurora Pujol, Erik A. Eklund, Darius Ebrahimi-Fakhari

**Affiliations:** 1 Movement Disorders Program, Boston Children’s Hospital, Boston, Massachusetts, USA.; 2 Neurometabolic Diseases, IDIBELL, Barcelona, Spain.; 3 CIBERER, Instituto de Salud Carlos III, Madrid, Spain.; 4 RSZ Translational Neuroscience Center, Boston Children’s Hospital, Boston, Massachusetts, USA.; 5 Hussman Institute for Human Genomics, University of Miami, Miami, Florida, USA.; 6 ICREA, Barcelona, Spain.; 7 Department of Pediatrics, Lund University, Lund, Sweden.

**Keywords:** Genetics, Neuroscience, Clinical practice, Movement disorders, Neurodegeneration

**To the Editor:** The hereditary spastic paraplegias (HSPs) are a group of more than 80 neurogenetic disorders that share the feature of progressive lower limb spasticity. Biallelic loss-of-function variants in the RINT1 gene have been implicated in acute liver failure in the pediatric population (1) and were recently described to lead to a complex form of HSP in 3 children with early-onset spastic paraplegia, ataxia, optic nerve hypoplasia with significant vision impairment, dysmorphic features, and a thin corpus callosum (2). We would like to add a fourth case due to what we believe are novel RINT1 variants presenting with a largely “pure” form of HSP (Supplemental Table 1; supplemental material available online with this article; https://doi.org/10.1172/JCI178919DS1). Methods are detailed in Supplemental File 1.

The patient is a 4-year-old girl, who was 2.5 years old at the time of initial referral. She was born after an uncomplicated pregnancy and delivery to nonconsanguineous healthy parents of Swedish heritage. Family history was unremarkable. No dysmorphic features were identified. While she met all early developmental milestones appropriately, first concerns arose when she was found to have progressive in-toeing and toe walking, starting at around 15 months of age (Supplemental Video 1 and Supplemental File 1). Symptoms rapidly progressed, with a decline in gait and frequent falls, leading her to stop walking at 18 months. She regained the ability to walk with assistance at 20 months of age (Supplemental Video 1). At 3.5 years, her exam was notable for distal lower extremity spasticity (Modified Ashworth Scale 3 in gastrocnemius and soleus bilaterally) and weakness, prominent pyramidal signs, and a spastic-ataxic gait (Supplemental Video 1), necessitating ankle-foot orthoses for walking and making running or climbing stairs impossible.

Now at 4 years of age, clinical features have remained stable, with no new manifestations or progression. Notably absent are features of complex HSP such as delays in speech and cognitive domains. Brain and spine MR imaging, including MR spectroscopy, liver function tests, and routine laboratory studies were normal. Clinical exome sequencing was nondiagnostic. Research trio genome sequencing revealed rare compound heterozygous truncating variants in RINT1 (NM_021930.6: c.1501C>T, p.Arg501Ter; c.1671_1671+2del, p.Val557del). Direct gene sequencing of the proband and parents confirmed these variants in trans. Both variants are observed at low frequencies in gnomAD 4.0 (minor allele frequency 1.098 × 10–5 and 1.602 × 10–5; https://gnomad.broadinstitute.org/). The nonsense variant (c.1501C>T, p.Arg501Ter) was predicted to result in premature termination and nonsense-mediated decay. The 3-bp deletion (c.1671_1671+2del, p.Val557del) had been previously identified in 2 individuals by 2 diagnostic laboratories and classified as a variant of uncertain significance (ClinVar ID: 224920). No phenotypic information was available. This variant was predicted to maintain the reading frame and preserve the canonical splice junction, moving the splice donor site 2 bp upstream (SpliceAI donor loss 0.98 at 1 bp, donor gain 0.85 at –2 bp) and to result in a mutant protein missing a single amino acid at p.Val557del (Figure 1A). RNA sequencing in the patient’s fibroblasts confirmed these predictions; at the splice junction associated with the 3-bp deletion, loss of the codon for Val557 was demonstrated (Supplemental Figure 1A). The absence of transcripts containing the codon for Val557 also supported the prediction that the transcripts from c.1501C>T, p.Arg501Ter indeed undergo nonsense-mediated decay. Overall, these data indicate that the RINT1 variants in our patient allow for residual expression of a mutant protein, p.Val557del (Supplemental Figure 1B), which contrasts with variants reported by Launay et al. (2).

To determine the functional impact of these mutations, further experiments in patient fibroblasts were conducted. Protein levels of RINT1 were decreased compared with control samples, similar to results in patients P1 and P3 reported by Launay et al. (2) (Figure 1, B and C). Plasma lipidomic profiles showed reduced levels of diacylglycerol and cholesterol esters, as well as elevated levels of phosphatidylcholine, phosphatidylethanolamine, and phosphatidylserine (Figure 1D). The free cholesterol–to–cholesterol ester ratio was significantly reduced in patient cells, suggesting a higher degree of cholesterol esterification (Figure 1D). Consistent with an overall increase in phospholipid levels, the concentration of lysophospholipid derivatives was decreased (Figure 1E), supporting the hypothesis that RINT1 deficiency leads to a shift toward the use of diacylglycerols to synthesize phospholipids and an inhibition of the Lands cycle. Taken together, these findings support a common molecular mechanism in biallelic variants in RINT1; they alter NRZ-complex (composed of NBAS, RINT1, and ZW10) function, leading to reduced triglyceride and diglyceride synthesis, impaired cholesterol turnover, reduced phosphatidylcholine/phosphatidylserine ratios, and inhibition of the Lands cycle.

Following the molecular diagnosis of RINT1 deficiency in our patient, additional investigations, including repeat liver function tests and a hepatic ultrasound, revealed no abnormalities. Clinically, a concern for cortical visual impairment was raised, but a detailed ophthalmological assessment (including optical coherence tomography and electroretinogram) was normal. The proband will undergo annual visits with an ophthalmologist and hepatologist, and parents have been instructed about symptoms of liver dysfunction and precautions during fever/illness.

Our case illustrates that biallelic variants in RINT1 should be considered in children presenting with pure and complex HSP (Supplemental Table 1), many of whom might be initially misdiagnosed as having cerebral palsy. A pattern of relatively normal early motor development followed by rapid onset and progression of lower limb spasticity in early childhood is uncommon among the recessive forms of HSP. The exon-intron boundaries around intron 11 may represent a mutational hotspot, given variation at c.1671+2 and c.1672-1 in all reported cases thus far. A prompt diagnosis is important, as this allows for monitoring for liver dysfunction during periods of metabolic stress (fever or infection) and avoidance of hepatotoxic agents, as well as genetic counseling and monitoring of siblings. Future research in prospective longitudinal natural history studies (ClinicalTrials.gov NCT04712812) is needed to delineate the full phenotypic spectrum of RINT1-associated disease and possible genotype-phenotype correlations.

## Supplementary Material

Supplemental data

Unedited blot and gel images

Supplemental video 1

Supporting data values

## Figures and Tables

**Figure 1:**
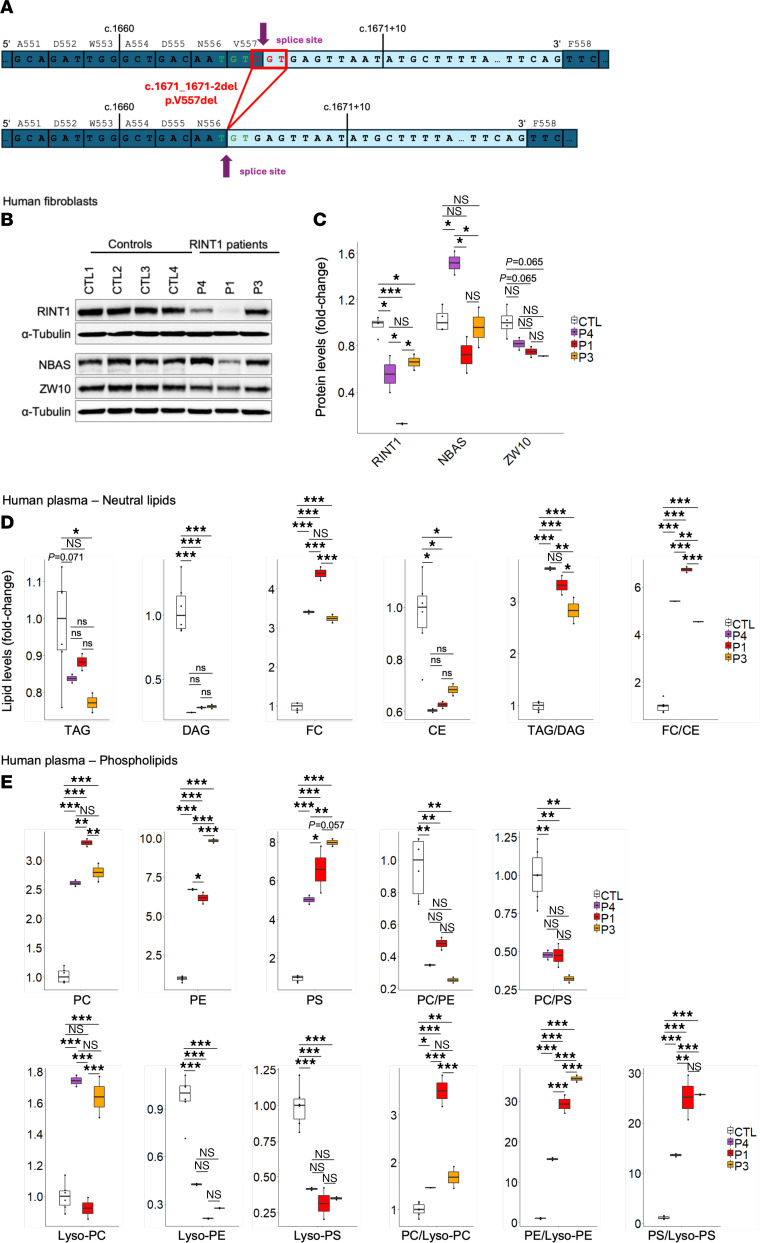
(**A**) Representation of the identified splice site 2 bp upstream because of c.1671_1671+2del. (**B** and **C**) Western blot analysis of NBAS, RINT1, and ZW10 and their quantification in fibroblasts from controls (CTL, *n* = 4), the patient (P4), and the previously published patients (P1 and P3). Lipidomic analysis of (**D**) glycerides and (**E**) phospholipids in plasma from controls (CTL, *n* = 6), the patient (P4), and the previously published patients (P1 and P3). All experiments were done in biological duplicates. Data are presented as box-and-whisker plots (median, interquartile interval, minimum, maximum). *P < 0.05; **P < 0.01; ***P < 0.001, 2-tailed Student’s *t* test. CE, cholesteryl esters; DAG, diacylglycerides; FC, free cholesterol; Lyso-PC, lysophosphatidylcholine; Lyso-PE, lysophosphatidylethanolamine; Lyso-PS, lysophosphatidylserine; PC, phosphatidylcholine; PE, phosphatidylethanolamine; PS, phosphatidylserine; TAG, triacylglycerides.

## Version 1 07/11/2024

In-Press Preview

## Version 2 09/03/2024

Electronic publication
