# Supplementary material for: Biallelic variants in RINT1 present as early-onset pure hereditary spastic paraplegia
Source: J Clin Invest. 2024 Jul 11;134(17):e178919. doi: 10.1172/JCI178919 (PMC11364375; doi:10.1172/JCI178919)
Supplement: Supplemental data [file jci-134-178919-s027.pdf]

SUPPLEMENTARY FILE 1.

PATIENT

This patient was identified through our HSP Genomic Sequencing Initiative (NCT05354622), part of the Children's Rare Disease Cohorts (1) at Boston Children's Hospital (IRB-P00039630). Written consent was obtained.

Supplementary Video 1: The patient's gait at different ages illustrating the rapid progression of spastic paraparesis. At 14-months there is no evidence of spasticity, and the gait is age-appropriate, at 21 months progressive toe walking and distal spasticity are evident, at 24 months and 29 months the gait is spastic-ataxic with frequent falls. This remains largely stable at 31 months.

MATERIALS AND METHODS

Sex as a biological variable

This report details findings in a single female patient with bi-allelic *RINT1* variants. The findings are expected to be relevant for female and male individuals.

Fibroblast cell culture

Fibroblasts were derived from the proband using a standard 2-mm skin punch biopsy. Fibroblasts were cultured and maintained as previously described (2). Cells were maintained in DMEM (Corning #10-013-CV) supplemented with 10% FBS (Life Technologies #10439-024), penicillin 100U/ml and streptomycin 100μg/ml (Gibco, #15140122), L-Glutamine (Thermo Fisher Scientific #25-030-08) with final concentration of 2mM, incubated at 37°C and 5 % CO_2. Cells were passed every 2-3 days at roughly 80 % confluency. For RNA sequencing, the cells were collected and pelleted by centrifugation, followed by snap-freezing in liquid nitrogen. RNA extraction, library preparation and sequencing were done at Azenta Life Sciences (South Plainfield, NJ, USA).

Whole genome sequencing and analysis

Following consent, blood was collected using EDTA tubes following. DNA isolation and trio short-read whole genome sequencing was performed at GeneDX (Gaithersburg, MD, USA) using the Illumina platform. Raw data underwent quality control, alignment, and variant calling using the DRAGEN platform and GATK guidelines. Subsequently, single nucleotide variants (SNV)s and small InDels were analyzed using the Emedgene platform, while copy number variations (CNVs) and other structural variants (SVs) were investigated using SequenceMiner. Variants were prioritized according to i) population allele frequency (maximal allele frequency, allele count, and number of homozygotes in gnomAD 4.0 (small variants) or Database of Genomic Variants (SVs), ii) previous ACMG classification reported to ClinVar, if any, iii) mode of inheritance and predictions of dosage sensitivity (ClinGen), iv) previous gene-phenotype

associations, v) gene constraint scores from gnomAD 4.0, vi) predictions of deleteriousness from tools such as CADD, REVEL, SpliceAI, vii) protein function, and viii) tissue expression. Potentially diagnostic variants were confirmed following CLIA guidelines by GeneDx. Whole genome sequencing data have been deposited to GEO (https://www.ncbi.nlm.nih.gov/geo/).

RNA sequencing and analysis

RNA samples were quantified using a Qubit 4 Fluorometer (Life Technologies), and RNA integrity was checked using Agilent TapeS-tation 4200 (Agilent Technologies). RNA sequencing libraries were prepared using the NEBNext Ultra II RNA Library Prep Kit for Illumina using the manufacturer's instructions (New England Biolabs). Briefly, mRNAs were initially enriched with Oligod(T) beads. Enriched mRNAs were fragmented for 15 min at 94 °C. First-strand and second-strand cDNA were subsequently synthesized. cDNA fragments were end- repaired and adenylated at 3' ends, and universal adapters were ligated to cDNA fragments, followed by index addition and library enrichment by PCR with limited cycles. The sequencing library was validated on the Agilent TapeStation (Agilent Technologies) and quantified using Qubit 4 Fluorometer (Invitrogen) as well as by quantitative PCR (KAPA Bio- systems). The sequencing libraries were clustered on 3 lanes of flowcell. After clustering, the flowcell was loaded on the Illumina instrument (HiSeq 4000 or equivalent) according to the manufacturer's instructions. The samples were sequenced using a 2x150bp Paired End (PE) configuration. Image analysis and base calling were conducted by the Control software. Raw sequence data (.bcl files) generated by the sequencer were converted into fastq files and de-multiplexed using Illumina's bcl2fastq 2.17 software. One mismatch was allowed for index sequence identification.

Using Trimmomatic, sequencing reads were processed to remove adapter sequences. Subsequently, reads were aligned to the human reference genome GRCh38 using STAR aligner v2.7.3a. SAMtools v.1.13 was used for indexing and Picard to mark duplicates. Post-alignment, normalized bigWig files were generated for visualization, and quality control was performed using RSeQC. The aligned reads were visualized using IGV (version 2.17) (3), focusing on variants of interests. Sashimi plots were generated for exon 11 alongside the start of exon 12.

Transcriptome data have been deposited to GEO (https://www.ncbi.nlm.nih.gov/geo/).

Western blot

Human fibroblasts were homogenized in RIPA buffer (150 mM NaCl, 1% Nonidet P40, 0.5% sodium deoxycholate, 0.1% SDS, 50 mM Tris, pH 8.0), sonicated for 1 minute at 4ºC, centrifuged for 10 minutes at 1,000 x g, mixed with 4X NuPAGE LDS Sample Buffer (Invitrogen), and heated for 10 minutes at 70ºC. Protein amounts were quantified using a BCA protein assay kit (Thermo Fisher Scientific). Protein samples (25 µg) were subjected to polyacrylamide gel electrophoresis for 1 hour at 120 V in NuPAGE MOPS SDS Running Buffer (Invitrogen) supplemented with 5 mM sodium bisulfite (Merck, 243973). Proteins were transferred to nitrocellulose membranes using an iBlot 2 Gel Transfer Device (Invitrogen). After blocking with 5% bovine serum albumin (BSA, Merck) in 0.05% TBS-Tween (TBS-T) for 1 hour at room temperature, membranes were incubated with primary antibodies overnight at 4°C. Following incubation with secondary antibodies for 1 hour at room temperature, proteins were detected with a Chemidoc™ Touch

Imaging System (BioRad). Bands were quantified with ImageLab (BioRad). The following antibodies were employed: anti-RINT1 (Merck; catalog HPA019875; 1:500), anti-NBAS (Abcam; catalog ab122370; 1:500), anti-ZW10 (Proteintech; catalog 24561-1-AP; 1:500), anti-α-Tubulin (Abcam; catalog ab7291; 1:10,000), polyclonal goat anti-rabbit (DakoCytomation; catalog P0448; 1;10,000), and polyclonal goat anti-mouse (DakoCytomation; catalog P0447; 1:30,000).

Lipidomic profiling

Human whole blood was centrifuged at 400 x g for 30 minutes using a gradient of Histopaque (Merck) to separate plasma, erythrocytes, and peripheral blood mononuclear cells (PBMC). Lipids were analyzed as described (4, 5) with minor modifications. In detail, for phospholipids and neutral lipids, a total of 750 µl of a methanol-chloroform (1:2, vol/vol) solution containing internal standards (16:0 D31_18:1 phosphatidylcholine, 16:0 D31_18:1 phosphatidylethanolamine, 16:0 D31-18:1 phosphatidylserine, 17:0 lysophosphatidylcholine, 17:1 lysophosphatidylethanolamine, 17:1 lysophosphatidylserine, 17:0 D5_17:0 diacylglyceride, 17:0/17:0/17:0 triacylglyceride and C17:0 cholesteryl ester, 0.2 nmol each, from Avanti Polar Lipids) were added to 0.002 ml plasma. Samples were vortexed and sonicated until they appeared dispersed and extracted at 48ºC overnight. The samples were then evaporated and transferred to 1.5 ml Eppendorf tubes after adding 0.5 ml of methanol and let to evaporate to dryness. Before analysis, 150 µl of methanol were added to the samples, centrifuged at 13,000 x g for 3 minutes, and 130 µl of the supernatants were transferred to ultra-performance liquid chromatography (UPLC) vials for injection and analysis.

All lipids were analyzed by liquid chromatography-high resolution mass spectrometry (LC-HRMS) using an Aqcuity ultra high-performance liquid chromatography (UHPLC) system (Waters, USA) connected to a Time of Flight (LCT Premier XE) Detector. Full scan spectra from 50 to 1,800 Da were acquired, and individual spectra were summed to produce data points each of 0.2 seconds. Mass accuracy at a resolving power of 10,000 and reproducibility were maintained using an independent reference spray via the LockSpray interference. Lipid extracts were injected onto an Acquity UHPLC BEH C8 column (1.7 µm particle size, 100 mm x 2.1 mm, Waters, Ireland) at a flow rate of 0.3 ml/min and a column temperature of 30ºC. The mobile phases were methanol with 2 mM ammonium formate and 0.2% formic acid (A)/water with 2mM ammonium formate and 0.2% formic acid (B).

Positive identification of compounds was based on the accurate mass measurement with an error B5 ppm and its LC retention time, compared with that of a standard (92%). Quantification was carried out using the extracted ion chromatogram of each compound, using 50 mDa windows. As indicated above, the linear dynamic range was determined by injecting mixtures of internal and natural standards. Since standards for all identified lipids were not available, the amounts of lipids are given as pmol equivalents relative to each specific standard.

Statistical analysis

Statistical significance was assessed through pairwise comparisons using t-tests, corrected for multiple testing with the Benjamini-Hochberg false discovery rate method, and was considered meaningful at p-value<0.05. Statistical analyses were performed using R software.

Study approval

This study was approved by the institutional review board at Boston Children's Hospital, Boston, MA (IRB-P00039630). Written consent was obtained.

Data availability

Data can be obtained from the corresponding author upon reasonable request.

AUTHOR CONTRIBUTIONS

Designing the research study: D.E.F.; acquiring data: V.Q., L.P.-S., A.Sv, A.T., H.K., U.Z., D.R. A.Sa., M.C., L.S., A.P., E.E., D.E.F.; analyzing data: V.Q., L.P.-S., A.Sv, A.T., U.Z., A.Sa., M.D., S.Z., M.C., L.S., A.P., E.E., D.E.F.; writing the manuscript: V.Q., L.P.-S., A.T., H.K., U.Z., L.S., D.E.F.; revising the manuscript: all authors.

ACKNOWLEDGMENT

The authors thank the patient and her family for supporting this research. This project was supported by the Boston Children's Hospital Children's Rare Disease Cohorts (CRDC). Research in the Ebrahimi-Fakhari Laboratory is supported by the Spastic Paraplegia Foundation, the Boston Children's Hospital Translational Research Program, and the National Institutes of Health / National Institute of Neurological Disorders and Stroke (K08NS123552-01). Vicente Quiroz is supported by a fellowship from the International Parkinson and Movement Disorder Society. Luca Schierbaum is supported by a fellowship from the German Research Foundation (536105452). The authors acknowledge the Boston Children's Hospital's High-Performance Computing Resources BCH HPC Clusters Enkefalos 3 (E3) made available for conducting the research reported in this publication. Work at the Neurometabolic Diseases Lab, IDIBELL, was supported by the Centre for Biomedical Research on Rare Diseases (CIBERER) (ACCI19-759), the URDCat programme (PERIS SLT002/16/00174), the IMPACT Genomica project, the Hesperia Foundation, the Secretariat for Universities and Research of the Ministry of Business and Knowledge of the Government of Catalonia (2021SGR00899), La Marató de TV3' Foundation (202006-30) to AP, and the Association ASL-HSP for resarch on Spastic Paraplegias to AP.

References:

1. Rockowitz S, LeCompte N, Carmack M, Quitadamo A, Wang L, Park M, et al. Children's rare disease cohorts: an integrative research and clinical genomics initiative. *NPJ Genom Med.* 2020;5:29.
2. Saffari A, Brechmann B, Boger C, Saber WA, Jumo H, Whye D, et al. High-content screening identifies a small molecule that restores AP-4-dependent protein trafficking in neuronal models of AP-4-associated hereditary spastic paraplegia. *Nat Commun.* 2024;15(1):584.
3. Robinson JT, Thorvaldsdottir H, Wenger AM, Zehir A, and Mesirov JP. Variant Review with the Integrative Genomics Viewer. *Cancer Res.* 2017;77(21):e31-e4.
4. Simbari F, McCaskill J, Coakley G, Millar M, Maizels RM, Fabriás G, et al. Plasmalogen enrichment in exosomes secreted by a nematode parasite versus those derived from its mouse host: implications for exosome stability and biology. *J Extracell Vesicles.* 2016;5:30741.
5. Barbacini P, Casas J, Torretta E, Capitanio D, Maccallini G, Hirschler V, et al. Regulation of Serum Sphingolipids in Andean Children Born and Living at High Altitude (3775 m). *Int J Mol Sci.* 2019;20(11).

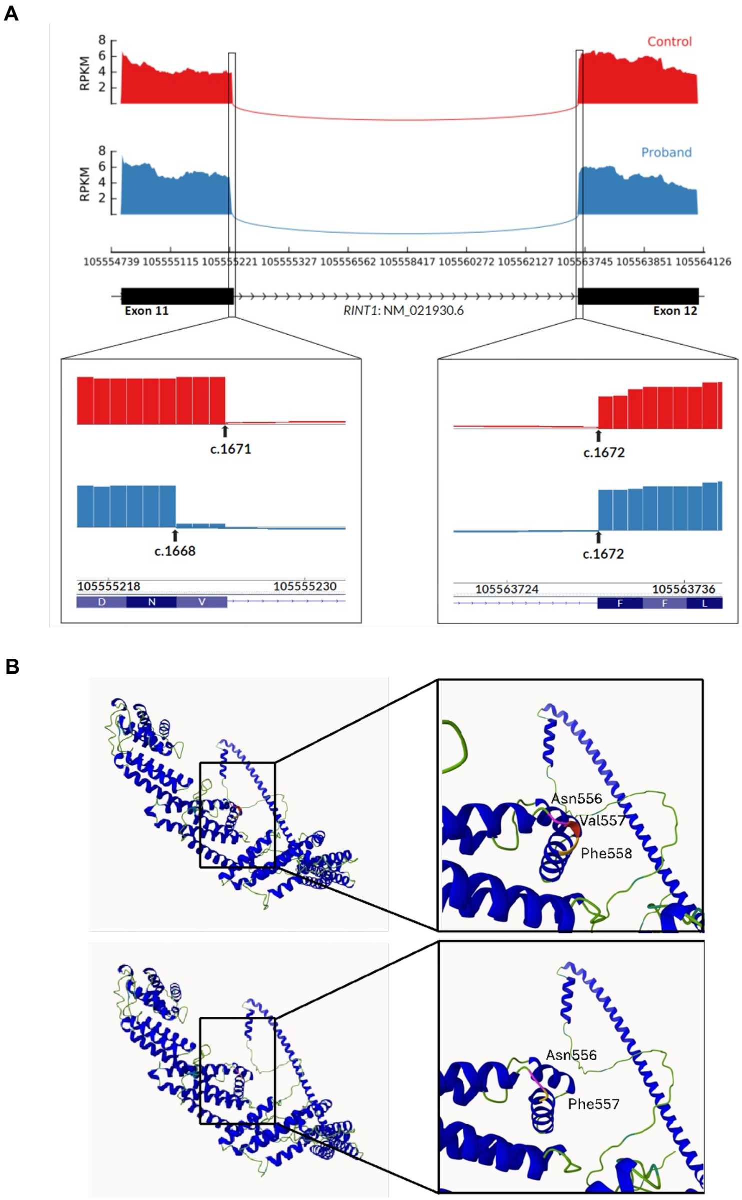

Supplementary Figure 1.

(A) RNA sequencing shows that all reads splice correctly to the original acceptor site, despite the new donor site being 2 base pairs upstream, continuing in-frame, similar to control. The complete absence of transcripts containing the codon for Val557 also supported the prediction that the transcripts from c.1501C>T, p.(Arg501Ter) indeed undergo nonsense-mediated decay. **(B)** Predicted three-dimensional structures of wildtype RINT1 protein (upper panel, Uniprot entry #Q6NUQ) and the mutant protein carrying the c.1671_1672+2del variant (lower panel), using SwissModel and Molstar. Alpha helices are colored dark blue, beta sheets light blue, and side chains green. The deleted amino acid Val557 is indicated in red, adjacent amino acid Asn556 in pink, and Phe558 in yellow.

Supplementary Table 1. Summary of all published cases of *RINT1*-associated hereditary spastic paraplegia

Patient	P1 (Launay et al.)	P2 (Launay et al.)	P3 (Launay et al.)	Case Presented
Maternal *RINT1* Variant	p.(Arg519Ter)	p.(Arg519Ter)	c.1672-1G>C p.(Phe558_Gln564del)	c.1501C>T p.(Arg501Ter)
Paternal *RINT1* Variant	c.1672-1G>A p.(Phe558_Gln564del)	c.1672-1G>A p.(Phe558_Gln564del)	c.1672-1G>C p.(Phe558_Gln564del)	c.1671_1671+2del p.(Val557del)
Sex	Male	Male	Female	Female
Self-assigned race (country of residence)	White (France)	White (France)	White (Spain)	White (Sweden)
Age of initial presentation	7 months	12 months	17 months	15 months
Age of most recent presentation	12 years	Died at 14 months	9 years	3.5 years
Development				
Motor development	Delayed	Normal	Delayed (walking at 20 months, instability)	Normal early motor milestones
Language acquisition	Delayed	N/A	Normal	Normal
Neurological Features				

Cognition estimated by a physician	Mild-moderate impairment	-	Normal, but has comprehension and abstract reasoning difficulties.	Cognitive, social and language development are appropriate for age
Muscle tone (age of onset)	Spastic paraparesis (18 months)	Normal	Spastic paraparesis (3 years)	Spastic paraparesis (18 months)
Cerebellar dysfunction (age of onset)	Dysmetria (6 years)	No	Ataxic gait (17 months) Fine postural tremor	Ataxic gait (18 months)
Behavior	Attention deficit disorder, low frustration tolerance, obsessive behaviors	-	Normal	Normal
MRI description	Posterior periventricular and external capsule T2 and FLAIR hyperintensities, thin corpus callosum, cerebellar and optic nerve atrophy. MRS: Increased peaks of	Normal	Normal	Normal brain and spine MRI. Normal MRS of the brain

	NAA and creatine, no increased lactate			
Other Features				
Hepatic Involvement	Acute hepatitis at 10 years	Fulminant acute hepatitis at 12 months	-	No hepatic involvement at age 3
Laboratory abnormalities	AST 340UI/L, ALT 3261UI/L, GGT 147UI/L. Normal lactate and NH3	Hypoglycemia, AST 7000UI/L, prothrombin time 5%, coagulation factor V 7%	-	No abnormalities reported
Dysmorphology	Anteverted nose, high-arched palate, low-set ears	Normal	Wide forehead, low-set ears	Normal
Other features	Umbilical hypoplasia, left adducted thumb, kyphosis	Vacuolation in renal tubules< sudden death at 14 months	Deterioration during fever episode at 3 years, genu valgum, Wormian bones	-

RINT1 transcript: NM_021930.6
