# Supplementary figures and images for: Biallelic variants in RINT1 present as early-onset pure hereditary spastic paraplegia
Source: J Clin Invest. 2024 Jul 11;134(17):e178919. doi: 10.1172/JCI178919 (PMC11364375; doi:10.1172/JCI178919)

Human fibroblasts

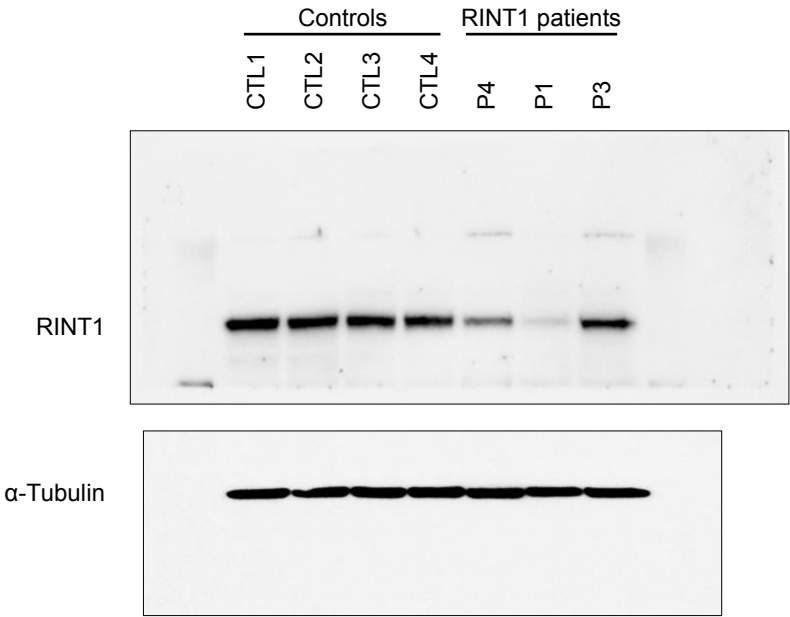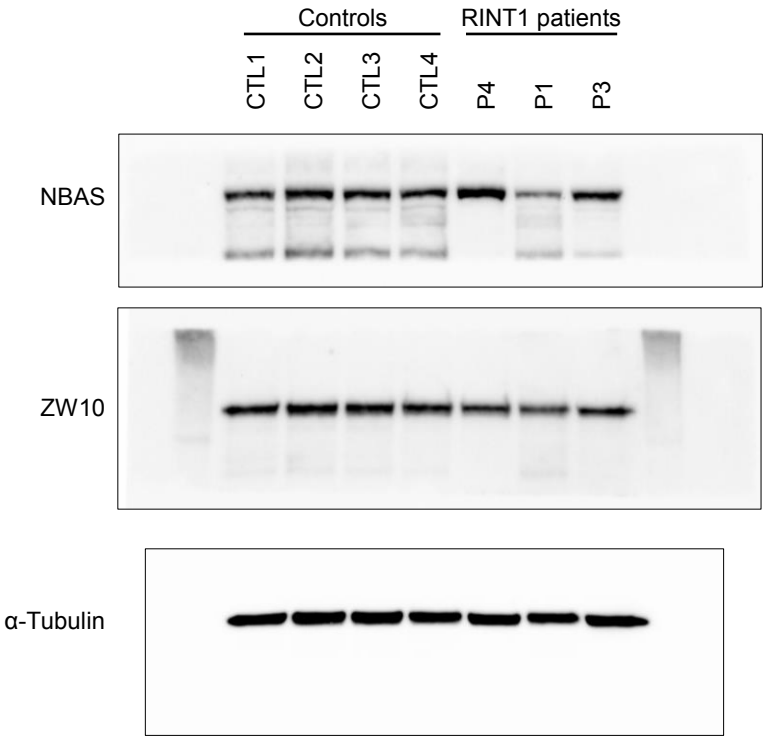

Supplement: Unedited blot and gel images [file jci-134-178919-s028.pdf]
